# Structure, Regulation, and Function of Linear and Circular Long Non-Coding RNAs

**DOI:** 10.3389/fgene.2020.00150

**Published:** 2020-03-03

**Authors:** Tao Qin, Juan Li, Ke-Qin Zhang

**Affiliations:** State Key Laboratory for Conservation and Utilization of Bio-Resources in Yunnan, Yunnan University, Kunming, China

**Keywords:** linear lncRNAs, circRNAs, structure, regulation, function

## Abstract

Long non-coding RNAs (lncRNAs), including linear lncRNAs and circular RNAs (circRNAs), exhibit a surprising range of structures. Linear lncRNAs and circRNAs are generated by different pathways. Linear lncRNAs perform functions that depend on their specific sequences, transcription, and DNA elements of their gene loci. In some cases, linear lncRNAs contain a short open reading frame encoding a peptide. circRNAs are covalently closed RNAs with tissue-specific and cell-specific expression patterns that have recently been extensively investigated. Pioneering work focusing on their biogenesis and functional characterization indicates that circRNAs regulate cell development via multiple mechanisms and play critical roles in the immune system. Furthermore, circRNAs in exosomes function on target cells. As with linear lncRNAs, specific circRNAs can also be translated. In this review, we summarize current understanding and highlight the diverse structure, regulation, and function of linear lncRNAs and circRNAs.

## Introduction

The central dogma of molecular biology describes the relationship between the informational macromolecules, DNA and RNA, and the transfer of the encoded information into proteins (Crick, 1970). However, more than 98% of the human genome is transcribed but only 2% of transcripts encode proteins (Wang et al., 2016; Kopp and Mendell, 2018). The transcripts that are not translated into proteins have been characterized as non-coding RNAs (ncRNAs). Based on their transcript size, they can be defined as small (≤200 nt) (small ncRNAs) and long ncRNAs (>200 nt) (lncRNAs), respectively. Small ncRNAs consist of transfer RNAs (tRNAs), small nucleic RNAs (snRNAs), small nucleolar RNAs (snoRNAs), microRNAs (miRNAs), piwi-interacting RNAs (piRNAs), and QDE-2-interacting RNAs (qiRNAs) (Madhani, 2013; Svendsen and Montgomery, 2018). These small ncRNAs participate in gene regulation at both transcriptional and post-transcriptional levels (Lee et al., 2009; Anderson and Ivanov, 2016; Czech and Hannon, 2016; Wang et al., 2016; Meng X. Y. et al., 2017; Xing et al., 2017; Shen et al., 2018). In contrast to small ncRNAs, lncRNAs, which were once thought to merely represent noise from imprecise transcription initiation, have been proposed to carry out diverse functions in cells (Mowel et al., 2017). Most well-characterized linear lncRNAs are transcribed by RNA polymerase II (Pol II) and are presumably capped, polyadenylated, and contain exon-exon splice junctions like mRNAs (Chen, 2016). However, the 3′ ends of a number of linear lncRNAs are formed in unusual ways (Chen, 2016). It seems that the unique end structures of linear lncRNAs protect their internal sequence and provide localization signals (Xing et al., 2017). Based on their genomic locations relative to adjacent protein-coding genes, lncRNAs are classified as sense, antisense, bidirectional, intronic, and intergenic lncRNAs (Wang et al., 2011; Chen, 2016; Wu et al., 2017; Kopp and Mendell, 2018).

Linear lncRNAs have diverse functions, including maintaining nuclear structure integrity (Clemson et al., 2009; Lei et al., 2013), positively or negatively regulating genes in *cis* or in *trans* by recruiting transcription factors or chromatin-modifying complexes to DNA targets in the nucleus (Long et al., 2017), acting as decoys to sequester RNA binding proteins (RBPs), or directly interacting with DNA (Chen, 2016; Cloutier et al., 2016). Linear lncRNAs in the cytoplasm serve as competing endogenous RNAs (ceRNAs) for miRNAs. In some cases, linear lncRNAs interact with RBPs to regulate signaling pathways (Fei et al., 2017; Jiang et al., 2018). Nevertheless, linear lncRNAs themselves do not perform sequence-specific functions but their loci are often the source of regulatory elements, such as enhancers and promoters. The process of linear lncRNAs transcription may impact the expression of nearby genes by recruiting specific protein factors (Ebisuya et al., 2008; Kopp and Mendell, 2018; Sanli et al., 2018). Like proteins, the functions of linear lncRNAs depend on their localization pattern in the nucleus and cytoplasm (Chen, 2016; Xing et al., 2017). Moreover, the short peptides produced from some specific linear lncRNAs are also functional (Anderson et al., 2015; Matsumoto et al., 2016; Nelson et al., 2016).

Recent studies suggest that some lncRNAs can form as a circle (circRNAs) and can function as a sponge to recruit miRNAs or transcriptional effectors to regulate target gene expression. Most circRNAs consist of one or more exons, termed extra-coding RNAs (ecRNAs), but some derive from the intron of the parent gene, such as circular intronic RNAs (ciRNAs) and intron retained circRNAs (exon-intron circRNAs, also known as EIciRNAs). circRNAs are more stable than linear ncRNAs because their circular structure cannot be degraded by most RNA decay machinery (Vicens and Westhof, 2014; Meng S. et al., 2017). The first circRNAs to be identified, *viroid*, was found in RNA viruses as early as 1976 (Sanger et al., 1976) and was then found in eukaryotes in 1979 (Hsu and Coca-Prados, 1979). Electron microscopy directly proved that circRNAs exist in eukaryotic cells (Hsu and Coca-Prados, 1979). Although circRNAs have attracted increasing attention, our understanding of their functions is still limited (Li X. et al., 2018). They appear to control brain function by titrating miRNAs (Piwecka et al., 2017), interacting with *RNA*-binding domains to influence cancer development (Fang et al., 2018), and protecting mRNAs from degradation (Zhu et al., 2019). They can also be biomarkers of cancer (Li Y. et al., 2015), ciRNAs and EIciRNAs are localized in the nucleus, where they promote the transcription of their parent genes (Li X. et al., 2018). Some specific circRNAs encode peptides (Legnini et al., 2017; Zhang et al., 2018), but internal ribosome entry site (IRES) elements might be necessary for this (Tatomer and Wilusz, 2017; Yun et al., 2017). Furthermore, recent work has shown that circRNAs may play critical roles in innate immune pathways (Cadena and Hur, 2017; Chen et al., 2017)

Although models of regulation are well established in many species, tissues, and cells types, the functions of linear lncRNAs and circRNAs remain elusive. Here, we highlight advances in our understanding of the multiple structures, functions, and regulation of linear lncRNAs and circRNAs.

## Structures of Linear lncRNAs

**Figure 1:**
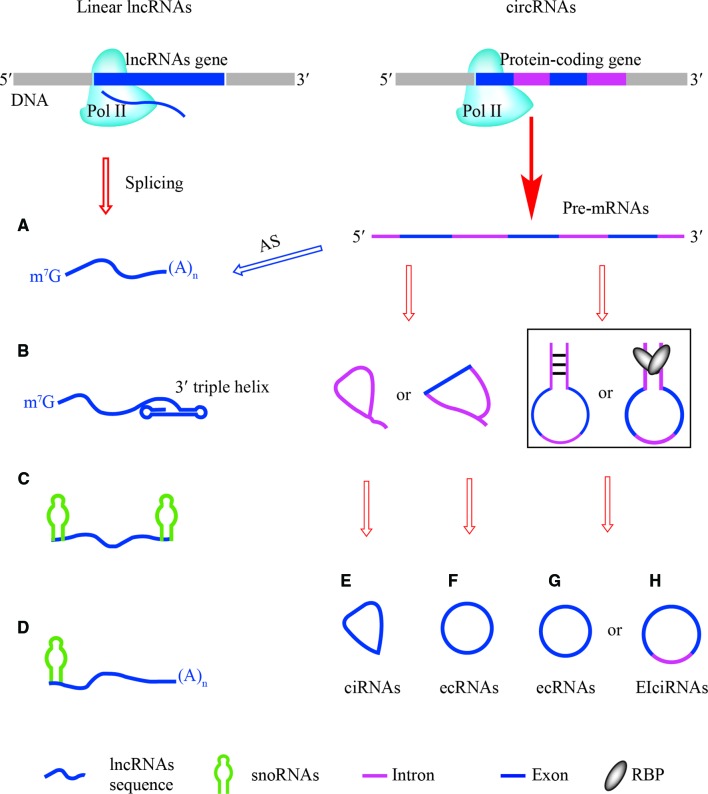
The structures of linear lncRNAs and circRNAs. **(A)** Linear lncRNAs with 5′ m^7^G and 3′ poly(A) ends are derived from specific loci (left) or alternative splicing of pre-mRNAs (right). **(B)** Linear lncRNAs with 3′ triple helix ends are alternatively processed by ribonuclease P (RNase P). **(C)** Small nucleolar RNAs (snoRNAs)-ended lncRNAs (sno-lncRNAs), their ends are capped by different structures of snoRNAs, BoxC/D-BoxC/D, BoxH/ACA-BoxH/ACA, BoxC/D-BoxH/ACA, and BoxH/ACA-BoxC/D. **(D)** The 5′ snoRNAs-ended and 3′-polyadenylated linear lncRNAs, their 5′ ends are capped by BoxC/D-poly(A) and BoxH/ACA-poly(A). **(E)** ciRNAs are derived from intron lariats of pre-mRNAs. **(F)** ecRNAs containing exon of pre-mRNAs are generated by internal back-splicing of lariats. **(G, H)** *cis*-acting and *trans*-acting factors are involved in the production of ecRNAs and EIciRNAs from pre-mRNAs.

The 5′ m^7^G cap and 3′ poly(A) tail are the hallmark structures of eukaryotic mRNAs and most annotated linear lncRNAs are transcribed from their own loci and are spliced just like mRNAs (Wu et al., 2017). However, linear lncRNAs also originate from pre-mRNAs as a result of alternative splicing (Grelet et al., 2017) (**Figure 1A**). The maturation and stabilization of many other linear lncRNAs are achieved through several non-canonical mechanisms that are highly associated with eukaryotic RNAs processing. For example, RNase P, which is best known for its function in tRNAs maturation, recognizes the tRNAs-like structure around the 3′ end of linear lncRNAs and generates a mature 3′ end with a U•A-U triple-helical structure (Wu et al., 2017) (**Figure 1B**). The 3′-end product is further cleaved by RNase Z to form MALAT1-associated small cytoplasmic RNAs (mascRNAs), whose function is still unknown (Wilusz et al., 2008).

Study of non-polyadenylated RNA transcripts in human cells revealed that many excised introns were longer than 200 nt, leading to the discovery of novel non-coding transcripts that lack 5′ caps and 3′ poly(A) tails but that have snoRNAs at both ends (Yin et al., 2012) (**Figure 1C**). A class of linear lncRNAs derived from the Prader-Willi Syndrome (PWS) region was identified and named sno-lncRNA, whose ends correspond to positions of intronic snoRNAs. These Box C/D sno-lncRNAs accumulate near their sites of synthesis and associate strongly with Fox family splicing regulators to alter splicing patterns (Yin et al., 2012). Likewise, one linear lncRNA called (snoRNA)-ended long non-coding RNA that enhances pre-rRNA transcription (*SLERT*) has a unique Box H/ACA snoRNA at both ends. Because *SLERT* is generated from the *TBRG4* gene locus, which is located at a distance from the nucleolus, these ends are critical to protect the *SLERT* internal sequence from degradation and for translocation to the nucleolus (Xing et al., 2017). Remarkably, six types of sno-lncRNAs have been described (Wu et al., 2016; Wu et al., 2017) and their ends are capped by BoxC/D-BoxC/D, BoxH/ACA-BoxH/ACA, BoxC/D-BoxH/ACA, BoxH/ACA-BoxC/D, BoxC/D-poly(A) (5′snoRNA capped and 3′polyadenylated), and BoxH/ACA-poly(A) (**Figures 1C, D**).

## Regulations of Linear lncRNAs

### Linear lncRNAs Regulate Gene Expression in *cis* or in *trans*

**Figure 2:**
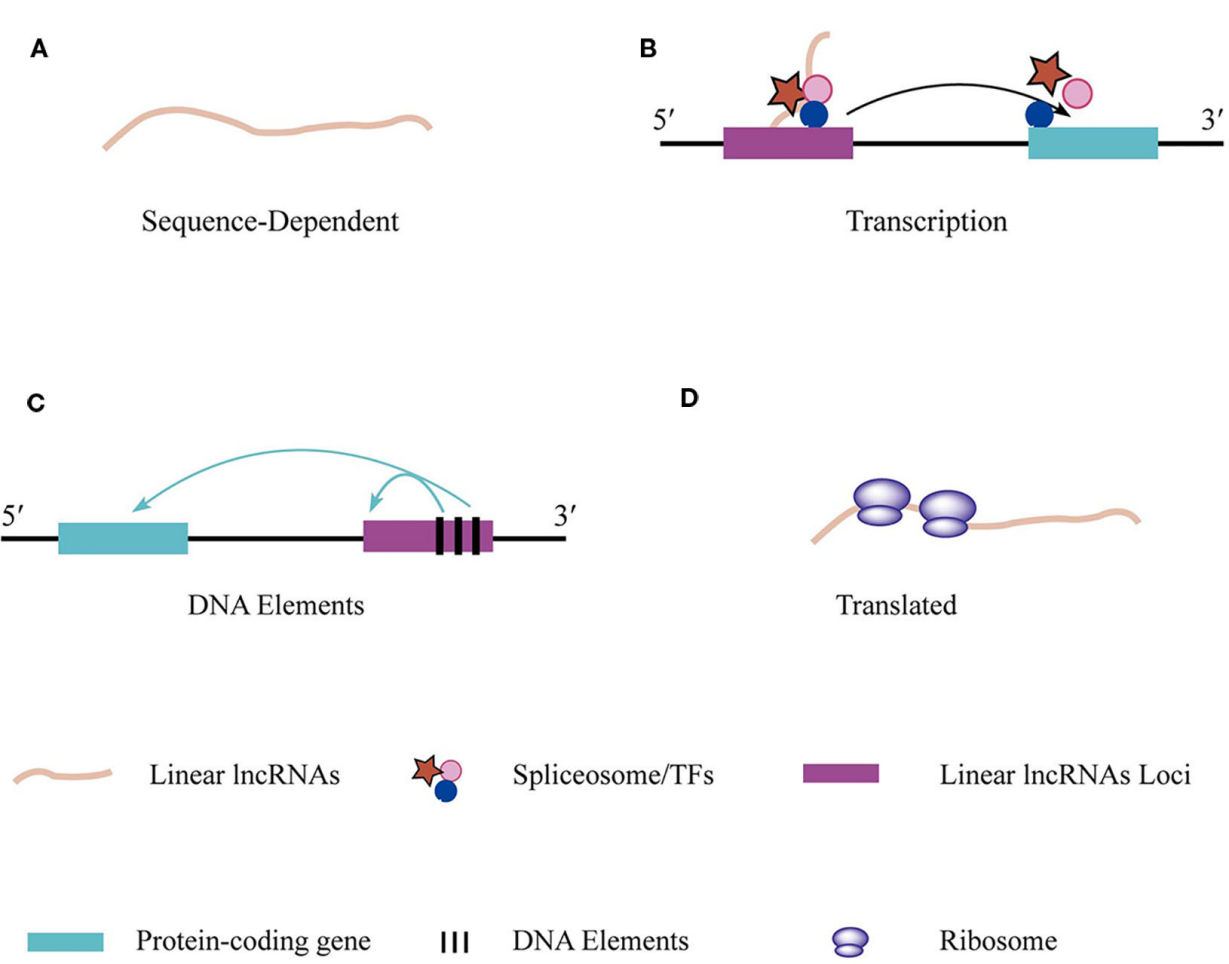
The regulations and functions of linear lncRNAs. **(A)** Linear lncRNAs perform function based on their unique nucleotide sequence which specifically bind to DNA, RNA, and RBPs or absorb miRNAs. **(B)** The transcriptional action of linear lncRNAs regulates adjacent gene expression. **(C)** DNA elements that embed in linear lncRNAs loci are able to regulate adjacent gene transcription **(D)** Some specific linear lncRNAs that contain open reading frame can be translated.

The functions of most linear lncRNAs depend on their sequence (**Figure 2A**). Various linear lncRNA functions are listed in **Table 1**. The most famous and well-established example of a *cis*-acting linear lncRNA is the X-inactive specific transcript, *Xist* (Penny et al., 1996; Cerase et al., 2015). In placental mammals, one of the two X chromosomes is transcriptionally silenced in the early embryo to provide dosage compensation. During X chromosome inactivation (Galupa and Heard, 2015), *Xist* is only transcribed from the inactivated chromosome. Despite the *Xist* lncRNA having been studied for several decades, its molecular functions are still highly debated (Cerase et al., 2015). In contrast, *cis*-acting linear lncRNAs are involved in dosage compensation in male *Drosophila melanogaster* by doubling the transcription of many X-linked genes (Gelbart and Kuroda, 2009). Activating DCCs (dosage compensation complexes) consist of two linear lncRNAs (roX1 and roX2 RNAs) and five male-specific-lethal (MSL) proteins (MSL1, MSL2, MSL3, the acetyltransferase, MOF, and the RNA helicase, MLE). DCCs only form in male flies because of the male-specific expression of the core MSL2 subunit and roX RNA (Maenner et al., 2013). Despite differing in size and having little sequence similarity, roX1 and roX2 are both thought to be scaffolds for the proper assembly of the MSL proteins. A series of conserved sequence motifs (GUUNUNCG) in the 3′ end of the roX RNA participate in the formation of a stable stem-loop structure (SLroX). The SLroX structure is important for roX RNA function (Maenner et al., 2013). The DCCs recognize the X chromosome through a limited number of “chromosomal entry” or “high-affinity” sites and the incorporation of roX RNA increases their interactions (Fang et al., 2008). Once DCCs are tethered to the active chromatin, lysine 16 of histone H4 (H4K16) is acetylated by MOF, leading to gene activation (Maenner et al., 2013).

**Table 1:** The identified linear lncRNAs functions.

Name	Origin	Mechanism	Function	References
*Xist*	Placental mammals	Sequence-dependent	Gene activation	(Penny et al., 1996)
roX1/roX2	*Drosophila melanogaster*	Sequence-dependent	Gene activation	(Gelbart and Kuroda, 2009)
*Linc-RAM*	Mouse	Sequence-dependent	Gene activation	(Yu et al., 2017)
*HOTAIR*	Human	Sequence-dependent	Gene inactivation	(Portoso et al., 2017)
^DRR^eRNA	Mouse	Sequence-dependent	Gene activation	(Li et al., 2016)
*lnc-DC*	Human	Sequence-dependent	Interacts with protein	(Pin et al., 2014)
*lncRNA-ACOD1*	Human	Sequence-dependent	Interacts with protein	(Wang et al., 2017)
*lnc-Lsm3b*	Human	Sequence-dependent	Decoy	(Jiang et al., 2018)
*Lnczc3h7a*	Human	Sequence-dependent	Scaffold	(Lin et al., 2019)
*H19*	Human	Sequence-dependent	miRNA sponge	(Kallen et al., 2013)
*Ftx*	Placental mammals	Transcription	Gene activation	(Furlan et al., 2018)
*Meg3*	Mouse	Transcription	Gene inactivation	(Sanli et al., 2018)
*PVT1*	Human	DNA elements	Gene activation	(Cho et al., 2018)
*Bendr*	Mammalian	DNA elements	Gene activation	(Engreitz et al., 2016)
*LINC00961*	Human	Can be translated	Produces a peptide	(Matsumoto et al., 2016)
*HOXB-AS3*	Primates	Can be translated	Produces a peptide	(Huang et al., 2017)

In contrast to *cis*-acting linear lncRNAs, some linear lncRNAs also positively or negatively regulate gene transcription at distant sites. One linear lncRNA named *Linc-RAM* (Linc-RNA Activator of Myogenesis) is specifically expressed in mouse skeletal muscle cells. *linc-RAM* is transcriptionally upregulated by MyoD and directly binds to MyoD, which promotes the assembly of the MyoD-Baf60c-Brg1 activation complex on specific regulatory elements of target genes (Yu et al., 2017). Some linear lncRNAs negatively regulate gene transcription in *trans*. The HOX antisense intergenic RNA, *HOTAIR* (2.2 kb), is a spliced and polyadenylated mammalian transcript derived from the *HOXC* locus, one of four HOX gene clusters (*HOXA*, *HOXB*, *HOXC*, and *HOXD*). Using chromatin isolation by RNA purification (ChIRP), *HOTAIR* was shown to interact with the *HOXD* cluster. Although newer methods are needed to further dissect the mechanism of *HOTAIR* function, it is thought to act as a scaffold that coordinates the recruitment of a chromatin-modifying complex to the distant *HOXD* locus, thereby establishing a repressed chromatin state (Portoso et al., 2017; Kopp and Mendell, 2018).

### New Insights Into eRNA Regulation

ncRNAs transcribed from active enhancers are known as eRNAs. eRNAs are an important component of transcriptional activation through their promotion of chromatin accessibility, Pol II recruitment, and enhancer-promoter contacts (Li et al., 2016). eRNAs perform their functions in *cis* or in *trans*. Upon enhancer activation, specific transcription factors binding to DNA motifs recruit transcription activators, such as histone acetyltransferases, CREB binding protein (CBP), and p300 (Goodman and Smolik, 2000; Creyghton et al., 2010). CBP and p300 are transcription co-activators that control the expression patterns of genes involved in cell growth, transformation, and development (Jin et al., 2014). Similar to polycomb repressive complex 2 (PRC2), CBP is also a chromatin-modifying enzyme whose activity can be regulated by direct binding to ncRNA (Cerase et al., 2015; Bose et al., 2017). CBP binds directly to a large number of eRNAs in cells. Steady-state histone acetyltransferase (HAT) assays revealed that the RNA binding region of CBP is the HAT domain. Briefly, eRNAs are transcribed from enhancers and act in *cis* to bind to the HAT domain of CBP to activate acetylation activity, which is required for the regulation of target genes (Bose et al., 2017).

Interestingly, the enhancer regions of mouse *MyoD*, which is located on chromosome 7, gives rise to at least two eRNAs. The core enhancer eRNA (^CE^RNA) influences the adjacent *MyoD* gene, whereas an eRNA named distal regulatory region (DDR) eRNA (^DRR^eRNA), which is transcribed from the enhancer of MyoD, acts in *trans* (Tsai et al., 2018). Chromatin isolation by RNA purification sequencing (ChIRP-seq) and single-molecule RNA fluorescence *in situ* hybridization (FISH) experiments indicated that ^DRR^eRNA co-localizes with nascent Myogenin transcripts, which are located on mouse chromosome 1. ^DRR^eRNA associates with the cohesin complex, which is required for cohesin chromatin recruitment and maintenance on chromosome 1, promoting the activation of the Myogenin gene (Tsai et al., 2018).

### Transcription of Linear lncRNAs Regulates Adjacent Gene Expression

Mammalian genomes are pervasively transcribed to produce enormous amounts of linear lncRNA. In addition to sequence-specific regulation, cross-talk exists between linear lncRNA expression and the expression of nearby genes (**Figure 2B**, **Table 1**). As described above, *Xist* promotes the process of X inactivation in female mammals. However, *Xist* expression needs to be tightly controlled by the X-inactivation center (Xic), which contains many linear lncRNA genes, such as *Linx*, *Jpx*, and *Ftx*. Linear lncRNA transcribed from *Ftx* or *Ftx*-embedded miRs had no impact on *Xist* transcription (Furlan et al., 2018). Interestingly, *Ftx* transcription was needed for *Xist* transcriptional activation at the onset of differentiation (Furlan et al., 2018). *Blustr* is a linear lncRNA located 5-kb upstream of the gene, *Sfmbt2*. Prematurely terminated transcription of *Blustr* or mutation of the first 5′ splice site of *Blustr* can abolish the expression of *Sfmbt2*, indicating that the *cis*-activating effect is associated with its transcription (Engreitz et al., 2016). The linear lncRNA, upper hand (*Uph*), provides an example of the translation of a non-coding RNA having a critical impact on the transcriptional activation of a nearby gene, *Hand2*. HAND2 is a transcription factor that controls the reprogramming of fibroblasts into cardiomyocytes. Termination of *Uph* transcription independently of its transcript resulted in the loss of *Hand2* expression in the mouse heart, which leads to embryonic lethality (Anderson et al., 2016).

Interestingly, the expression of linear lncRNAs may negatively regulate nearby genes. Delta-like-1 (*Dlk1*) encodes a ligand that inhibits Notch1 signaling and plays a role in placental development, nutrient metabolism, and adipocytosis (Sanli et al., 2018). The *Dlk1-Dio3* imprinted domain, which contains three protein-coding genes, *Dlk1* (also called *Pref1*), *Rtl1*, and *Dio3*, also expresses multiple ncRNAs, such as linear lncRNA, *Meg3* (maternally expressed gene 3, also called *Gtl2*), the C/D-box snoRNA cluster, *Rian*, the miRNA cluster, *Mirg*, and the Rtl1-antisense, *Rtl1as* (Sanli et al., 2018). In a hybrid embryonic stem cell system, *Dlk1* became imprinted and was involved in transcriptional upregulation on the paternal chromosome, while the maternal *Dlk1* gene remained poised for activation during neural differentiation. It has been postulated that the genes are repressed by one of the locus' ncRNAs. Both *Meg3* expression and the H3-Lys-27 methyltransferase, EZH2, prevent *Dlk1* activation in *cis* on the maternal chromosome. However, the *Meg3* linear lncRNA was partially retained in *cis* and overlaps with the maternal *Dlk1*. A future challenge is, therefore, to answer whether the *Meg3* linear lncRNA is involved in chromatin repression (Sanli et al., 2018).

### DNA Elements of Linear lncRNAs Regulate Adjacent Gene Expression

*Cis*-regulatory activity may involve DNA elements of a linear lncRNA locus, such as the promoter, but be independent of the linear lncRNA transcript or the transcription of linear lncRNA in general (**Figure 2C**, **Table 1**). Plasmacytoma variant translocation 1 (*PVT1*) was the first linear lncRNA identified in human Burkitt's lymphoma (Graham and Adams, 1986). *PVT1* and the myelocytomatosis (*MYC*) oncogenes are located some distance apart on 8q24 and the *PVT1*-encoded linear lncRNA and miRNA have oncogenic functions (Cho et al., 2018). Indeed, *PVT1* performs an oncogenic function by stabilizing the MYC protein (Tseng et al., 2014). However, silencing *PVT1* using CRISPR interference (CRISPRi) technology unexpectedly enhanced cell proliferation of glioblastoma cells and induced pluripotent stem cells. Furthermore, recurrent structural rearrangements of the *PVT1* locus disrupts *PVT1* transcription in cancer genomes, indicating that the *PVT1* locus has unknown regulatory mechanisms. These findings suggest that the *PVT1* locus harbors four intragenic enhancers of *MYC*, which promote *MYC* transcription. Furthermore, the competition for enhancers between the *PVT1* and *MYC* promoters is proposed to control cell development in glioblastoma cells and induced pluripotent stem cells independent of the *PVT1* linear lncRNA (Cho et al., 2018). As another example, the promoter of *Bendr* (*Bend4*-regulating effects not dependent on the RNA) has a regulatory function for the adjacent *Bend4* gene. Inserting a polyA signal into the first intron of *Bendr* had no effect on *Bend4* expression, indicating that the regulatory function was independent of *Bendr* RNA transcription. However, deletion of the ~750 bp *Bendr* promoter-proximal region reduced expression of the adjacent *Bend4* gene by 57%. Therefore, *cis* activation of *Bend4* requires the promoter of a nearby linear lncRNA (Engreitz et al., 2016; Kopp and Mendell, 2018).

## Functions of Linear lncRNAs

### Linear lncRNAs Interact Directly With Proteins, Serve as Decoys, and Act as Scaffolds

Proteins in the cytoplasm function through various mechanisms and, in some cases, their function is linked to a functional linear lncRNA (Pin et al., 2014). Early in 2014, *lnc-DC* was found to be exclusively expressed in conventional human dendritic cells (DCs) and to control human DC differentiation by directly binding to STAT3 (signal transducer and activator of transcription 3) in the cytoplasm (Pin et al., 2014). In another case, the metabolic enzyme, glutamic-oxaloacetic transaminase 2 (GOT2), was confirmed to be an *lncRNA-ACOD1* binding protein. *lncRNA-ACOD1* is induced by multiple viruses but is independent of type I interferon (IFN-I). Upon induction, *lncRNA-ACOD1* activates GOT2 and substantially changes cellular metabolism for the benefit of viruses. Down-regulation of *lncRNA-ACOD1* dramatically protects mouse and human cells from virus infection. It is considered to be a tactic by which viruses complete their life cycle and propagate (Wang et al., 2017).

Linear lncRNAs also serve as decoys to replace other kinds of RNA and then bind to host proteins. In mouse macrophages, retinoic acid-inducible gene I (*Rig1*) has the ability to bind to pathogenic RNA through its C-terminal domain (CTD) in the presence of ATP and to promote innate immunity (Jiang et al., 2018). RIGI recognizes double-stranded viral RNA (dsRNA) that has invaded cells and indirectly promotes the transcription of interferons. Strikingly, in addition to resisting virus RNA, interferons also induce the production of a linear lncRNA called *lnc-Lsm3b*. *lnc-Lsm3b* serves as decoy to replace viral RNA and it has been proposed that inhibition of RIG-I activation was probably because RIG-I oligomerization was prevented (Jiang et al., 2018). A similar model for myogenesis was reported by David Glass's group (Gong et al., 2015). One linear lncRNA, *LncMyoD*, located next to the *MyoD* gene, was directly activated by *MyoD* during myoblast differentiation, but did not bind to *MyoD*. RNA pull-down experiments identified IGF2-mRNA-binding protein 2 (IMP2) as the binding partner of *LncMyoD.* The increased level of *LncMyoD* serves as decoy to outcompete other mRNAs, such as *N-Ras* and *c-Myc*, blocking proliferation and creating a permissive state for differentiation (Gong et al., 2015).

In addition to these mechanisms of interacting with proteins, linear lncRNAs also regulate protein function by serving as a cytoplasmic scaffold. Recent attention has focused on an E3 ligase, tripartite motif 25 (TRIM25), a protein down-stream of RIGI. A linear lncRNA named *Lnczc3h7a* was detected in an RNA pull-down assay of RIGI. Overexpression of *Lnczc3h7a* increased the RIGI-TRIM25 interaction and K63-linked ubiquitination of RIGI in VSV-infected mouse fibroblast cells, but the interaction was absent in uninfected RAW264.7 cells. An RNA pull-down assay showed that *Lnczc3h7a* binds to the helicase of RIGI and the C-terminal SPRY domain within TRIM25. Individual-nucleotide-resolution cross-linking and immunoprecipitation experiments indicated that the *Lnczc3h7a* binding site of TRIM25 is around nucleotide 311, while the *Lnczc3h7a* binding sites of RIGI are around nucleotide 308 and 332. Altogether, *Lnczc3h7a* serves as a scaffold to facilitate the RIGI-TRIM25 interaction and to regulate their functions in response to virus infection (Lin et al., 2019).

### Linear lncRNAs Serve as Sponges for miRNAs

With the development of the competing endogenous RNAs (ceRNAs) hypothesis, a large number of linear lncRNAs that serve as miRNAs sponges were identified to play roles in regulating translation (Mousavi et al., 2014). The linear lncRNA, *MIR100HG*, and two *MIR100HG*-derived miRNAs, miR-100 and miR-125b, play important roles in the cetuximab resistance of cetuximab-sensitive CRC cells and head and neck squamous cell carcinoma cells lines (Lu et al., 2017). The transcription factor, GATA6, inhibits the production of the linear lncRNAs, *MIR100HG*, but one of the two miRNAs, miR-125b, provides feedback inhibition of GATA6 and relieves the repression. Thus, increased levels of *MIR100HG* produces more miR-100 and miR-125b, which coordinate to repress five Wnt/β-catenin negative regulators, resulting in increased Wnt/β-catenin signaling (Lu et al., 2017). The human linear lncRNAs, *H19*, is a ~2.3 kb, capped, spliced, and polyadenylated RNAs, predominantly located in the cytoplasm that is implicated in genetic disorders and cancer (Jia et al., 2018). However, the mechanisms by which *H19* regulates gene function remain elusive. *H19* can serve as a molecular sponge for miRNAs to modulate gene expression in the mouse myogenic C2C12 cell line. Bioinformatic analysis revealed that *H19* has one canonical and three non-canonical binding sites for the miRNAs, *let-7*, while *let-7* overexpression results in a differentiated myoblast phenotype. However, strongly induced *H19* acts as a sponge for *let-7* and hinders muscle differentiation (Kallen et al., 2013).

### Some Linear lncRNAs Encode Short Peptides

Deep-sequencing technologies have led to the identification of a large number of linear lncRNAs that lack obvious long protein-coding open reading frames (ORFs). However, some linear lncRNAs with putative small ORFs of less than 100 amino acids actually code for proteins that play important roles in cells (Anderson et al., 2015; Matsumoto et al., 2016; Nelson et al., 2016) (**Figure 2D**, **Table 1**). Furthermore, in some specific cases, linear lncRNAs possess dual functions that are dependent on both the linear lncRNA itself and proteins encoded by the linear lncRNA (Anderson et al., 2015; Yu et al., 2017).

Small regulatory polypeptide of amino acid response (SPAR) is a short, 90 amino acid peptide encoded in humans and mice by the linear lncRNA, *LINC00961*. SPAR plays an important role in muscle regeneration. SPAR contains a conserved transmembrane domain at its N terminus with its C terminus extending into the cytosol. Immunofluorescence staining showed that SPAR is localized to late endosomes/lysosomes. Further work revealed that this small peptide interacts with the lysosomal v-ATPase to negatively regulate mTORC1 activation by amino acids (Roberto et al., 2011). Using CRISPR/Cas9 engineering to knockout the SPAR peptide showed that down regulation of SPAR enables efficient activation of mTORC1 and promotes muscle regeneration (Matsumoto et al., 2016).

The Sarcoplasmic reticulum Ca^2+^-ATPase (SERCA) controls the release and reuptake of Ca^2+^ from the sarcoplasmic reticulum (SR). Myoregulin (MLN) and dwarf open reading frame (DWORF), which are 46 and 34 amino acids long, respectively, are two functional mammalian polypeptides encoded by linear lncRNAs that modulate the calcium pump, SERCA, and control muscle performance (Anderson et al., 2015; Nelson et al., 2016). MLN, phospholamban (PLN), and sarcolipin (SLN) share a similar structure and function and inhibit SERCA. In contrast to MLN, DWORF enhances SERCA activity by displacing the SERCA inhibitors MLN, PLN, and SLN (Nelson et al., 2016). Deletion of MLN in mice skeletal muscle enhances Ca^2+^ handling and improves exercise performance. Overexpression of DWORF in mouse cardiomyocytes increases peak Ca^2+^ transient amplitude and SR Ca^2+^ load during each cycle of contraction-relaxation, reducing the decay time constant of cytosolic Ca^2+^ (Nelson et al., 2016). Thus, MLN may have the opposite function to DWORF. Interestingly, in addition to encoding the peptide MLN, the linear lncRNA, linc-RAM, promotes myogenic differentiation by interacting with MyoD (Yu et al., 2017).

Small peptides encoded by putative linear lncRNAs can suppress cancer cell growth. Cancer cells, including colorectal cancer (CRC) cells, exhibit distinct metabolic reprogramming patterns, which support rapid proliferation (Israelsen and Vander Heiden, 2015). The glycolytic enzyme, pyruvate kinase M (PKM), plays an important role in this process (Mayumi et al., 2012). There are two PKM isoforms, PKM1 and PKM2, resulting from alternative splicing of the *PKM* pre-mRNA. PKM2 is almost universally re-expressed in cancer cells and supports the proliferation of cancer cells, whereas the adult isoform, PKM1, is universally expressed in normal cells. The *HOXB* cluster antisense RNA 3 (*HOXB-AS3*) gene is a linear lncRNA gene that has the ability to produce a conserved 53-amino acid peptide. This peptide modulates the alternative splicing of the *PKM* pre-mRNA to yield more PKM1, which suppresses CRC cell growth (Huang et al., 2017).

## Structures and Properties of circRNAs

Most annotated circRNAs originate from nucleolar pre-mRNAs, but some circRNAs are derived from mitochondria (Liu et al., 2019). Several mechanisms are associated with the biogenesis of circRNAs. ciRNAs are derived from the failure of intronic lariat debranching during canonical splicing (Zhang et al., 2013). Bioinformatic and experimental evidence indicate that the formation of ciRNAs depends on a consensus RNA motif that contains a 7-nt GU-rich element near the 5′ ss and an 11-nt C-rich element near the branch point (Zhang et al., 2013) (**Figure 1E**). Unlike linear mRNAs, which are mainly located in the cytoplasm, human ciRNAs are preferentially localized in the nucleus (Li X. et al., 2018). Additionally, lariats containing an exon can form during exon skipping and internal back-splicing allows the formation of extra-coding RNAs (Steven et al., 2015) (**Figure 1F**). The biogenesis of circRNAs in flies and humans is highly dependent on intronic sequences. RBPs also promote exon circularization by binding to sequences in the flanking introns (Zhang et al., 2013). In some cases, the biogenesis of circRNAs is influenced by a combination of *cis*-acting elements and *trans*-acting splicing factors (Kramer et al., 2015). Although the majority of circRNAs normally contain multiple exons (**Figure 1G**), alternative splicing allows intron retention within EIciRNAs, which have been found to remain in the nucleus (Zhang et al., 2014) (**Figure 1H**).

Some protein factors can disrupt *cis*-acting elements associated with circRNA biogenesis*. Alu* elements are repetitive elements that make up more than 10% of the human genome (De Koning et al., 2011) and which are involved in the biogenesis of endogenous circRNAs. However, the function of *Alu* elements is inhibited by nuclear RNA helicase, DHX9. DHX9 interacts directly with the interferon-inducible isoform of ADAR (p150) and disrupts circRNA biogenesis. The loss of DHX9 doubles the production of circRNAs (Aktaş et al., 2017).

Most circRNAs are exported to the cytoplasm from the nucleus, except for intron-containing circRNAs. Two proteins, spliceosome RNA helicase, DDX39B (also called DEAD box protein UAP56 or UAP56), and ATP-dependent RNA helicase, DDX39A (also called nuclear RNA helicase URH49 or URH49), are associated with transporting circRNAs. In humans, UAP56 exports circRNAs larger than 1,200 nucleotides whereas URH49 exports circRNAs smaller than 400 nucleotides (Huang et al., 2018).

So far, we know very little about the degradation mechanisms of circRNAs, but a few examples have been studied. For example, circRNA *CDR1as* (also known as ciRS-7) is degraded by the Argonaute 2 (AGO2) protein complex (Kopp and Mendell, 2018). The binding of miR-671 to *CDR1as* triggers AGO2-dependent cleavage of *CDR1as*. Evidence also shows that N^6^-methyladenosine (m^6^A) within circRNAs promotes their degradation (Park et al., 2019). Furthermore, the circRNA that binds to dsRNA-activated protein kinase (PKR) is degraded by RNase L (Liu et al., 2019).

## Regulations of circRNAs

### circRNAs in Exosomes

**Figure 3:**
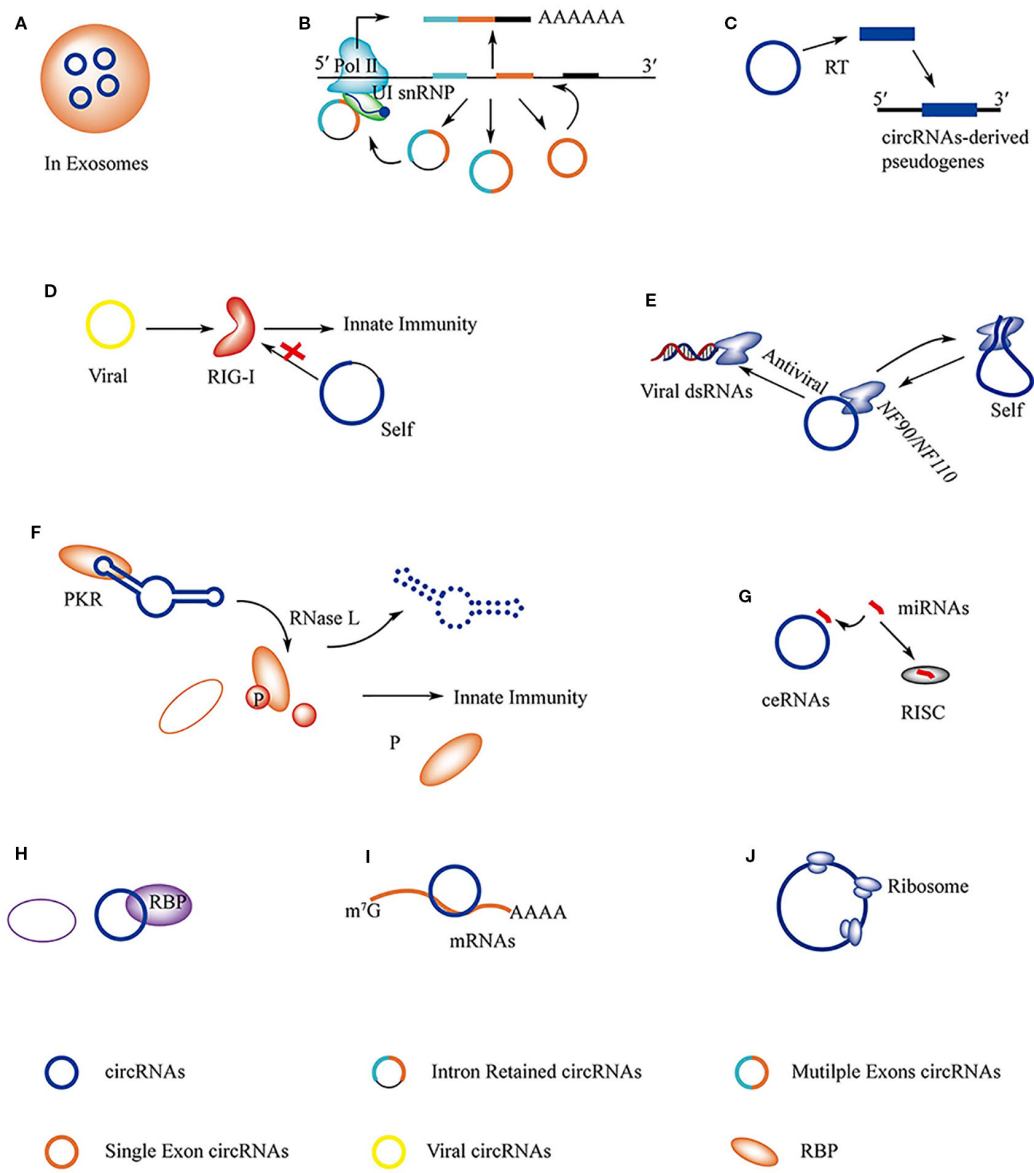
The regulations and functions of circRNAs. **(A)** circRNAs in exosomes can be transported from host cell to target cell where circRNAs serve as miRNAs sponges to regulate gene expression. **(B)** ciRNAs, EIciRNAs, and ecRNAs are able to regulate the expression of their maternal gene in nucleous. **(C)** Pseudogenes are derived from circRNAs. **(D)** RIG-I is able to distinguish viral circRNAs from self-originated ciRNAs and activate immune response. **(E)** Immune factor *NF90/NF110* has dual function that is associated with the biogenesis of circRNAs and innate immune response. **(F)** The structure of circRNAs play important roles in stabilizing PKR activity, the degradation of circRNAs by RNase L releases the activity of PKR and results in immune response. **(G)** circRNAs serve as miRNAs sponge in cell and regulate gene expression. **(H)** circRNAs are able to interact with RBPs directly and regulate its function; circRNAs serve as decoy to competitive binding to RBPs with other RNAs, like mRNAs and viral dsRNAs; circRNAs are found to act as scaffold to interact with RBPs. **(I)** circRNAs interact with mRNAs and protect it from degradation. **(J)** Specific circRNAs can be translated into protein.

Exosomes are small membrane vesicles of endocytic origin secreted by most cells types. Their cargos of proteins, mRNAs, and miRNAs modulate recipient cell behaviors. Recently, circRNAs have also been shown to be abundant in exosomes, where they may function as miRNA sponges (Li Y. et al., 2015) (**Figure 3A**). Indeed, circRNAs in exosomes secreted from adipocytes and cancer cells play important roles in regulating their target cells. In hepatocellular carcinoma (HCC) patients with higher body fat ratios, exosome circ-deubiquitination (*circ-DB*) from adipocytes is upregulated. Ubiquitin-specific protease 7 (USP7) is a deubiquitinating enzyme and a high level of USP7 is frequently found in HCC tissues. *circ-DB* absorbs miR-34a, which targets *USP7* mRNA and promotes the expression of USP7 (Zhang et al., 2019a). Interestingly, exosomal circRNAs derived from gastric cancer (GC) cells also function as sponges for miRNA. PR domain containing 16 (PRDM16), a zinc finger transcription factor, plays important roles in the browning of white adipose tissue (WAT) in GC patients. miR-133 has been proposed as an upstream regulator of PRDM16. The circRNA, *ciRS-133*, in exosomes derived from GC cells can sponge miR-133 and activate PRDM16 (Zhang et al., 2019b).

### circRNAs Regulate Maternal Gene Transcription

Most circRNAs are derived from the middle exons of protein-coding genes, and they can affect the splicing of their linear counterparts (**Figure 3B**). The second exon of the splicing factor, muscleblind (MBL/MBNL1), is circularized in flies and humans. Interestingly, the flanking introns of the second exon contain conserved muscleblind binding sites, which are bound by MBL. The MBL levels strongly affect the circulation and production of *circMbl.* Thus, the biogenesis of *circMbl* is strongly regulated by MBL and can function in gene regulation by competing with linear splicing (Reut et al., 2014).

*Arabidopsis circSEP3* is derived from exon 6 of SEPALLATA3 (SEP3). *circSEP3* binds strongly to its cognate DNA locus, forming an RNA:DNA hybrid, or R-loop. The formation of the R-loop results in transcriptional pausing and leads to the formation of alternatively spliced SEP3 mRNA with exon skipping, which in turn drives floral homeotic phenotypes (Conn et al., 2017). Another case is friend leukemia virus integration 1 (FLI1), a transcription factor that promotes tumor growth. Interestingly, a circRNA FLI1 exonic circular RNA (*FECR1*) derived from exons 4-2-3 of the *FLI1* pre-mRNA can bind to the FLI1 promoter and recruits demethylase TET1 to the promoter region of its own host gene (Chen et al., 2018).

Most circRNAs are located in the cytoplasm, but the intron lariats processed to ciRNAs or EIciRNAs are restricted to the nucleus in human cells. The nuclear retained circRNAs regulate transcription and splicing of their parent genes. The ciRNA, *ci-ankrd52*, is derived from the second intron of ankyrin repeat domain 52 (*ANKRD52*) and mainly accumulates in the nucleus, associating with the elongation Pol II machinery to positively regulate translation of its encoding gene (Zhang et al., 2013). The EIciRNA, *ElciEIF3J*, is predominantly localized in the nucleus, where it interacts with U1 snRNP via specific RNA-RNA interaction, promoting the transcription of their parental genes (Li Z. et al., 2015).

### Pseudogenes Derived From circRNAs

Pseudogenes are usually derived from the integration of reverse-transcribed linear mRNAs and it is estimated that about 10% of known gene loci in humans and mice are processed pseudogenes. Although circRNAs are speculated to be stable in cells, some circRNAs can be retro-transcribed and ultimately inserted back into the host genome as processed pseudogenes (**Figure 3C**). In contrast to linear mRNAs, circRNA-derived pseudogenes have an exon-exon junction in the reverse order (non-colinear), which allows for rearrangements of exons into the host genome. For example, 33 pseudogenes are predicted to be derived from *circRFWD2* because of the existence of the non-colinear exon 6-exon 2 junction sequence (Dong et al., 2016).

### circRNAs Are Involved in Innate Immune Responses

As discussed above, RIGI is necessary for activating innate immunity in response to viral infections. In addition to binding to viral dsRNA or specific self-produced linear lncRNAs, such as *lnc-Lsm3b* or *Lnczc3h7a*, RIGI also discriminates between exogenous and endogenous circRNAs. Delivery of purified *in vitro* generated circRNA to mammalian cells showed that RIGI is necessary for sensing foreign circRNAs, leading to the activation of innate immunity (Chen et al., 2017) (**Figure 3D**). Interestingly, RIGI does not recognize endogenous circRNAs. Using a human intron to express a foreign circRNA sequence abrogated immune activation confirming that RIGI discriminates between self- and foreign circRNAs by intron identity (Chen et al., 2017). However, the mechanism by which RIGI recognizes exogenous circRNAs remains unknown.

Protein factors involved in the biogenesis of circRNAs can act as immune factors (Li et al., 2017). Immune factors involved in circRNA formation were identified by combining immune factors with circRNA biogenesis and performing genome-wide siRNA screening. The double-stranded RNA-binding domain containing immune factors, *NF90/NF110*, were identified as a dual-function protein (Li et al., 2017). *NF90/NF110* promotes circRNA processing by stabilizing the flanking intronic RNA pairs in the nucleus and subsequently interacts with the mature circRNA to form the *NF90/NF110*-circRNA complex (circRNPs) in the cytoplasm. Upon viral infection, *NF90/NF110* is released from the circRNPs, resulting in an antiviral immune response (Cadena and Hur, 2017; Li et al., 2017) (**Figure 3E**).

Pioneering work by Liu et al. led to the discovery that many circRNAs play important roles in the autoimmune disease, systemic lupus erythematosus (SLE) (Liu et al., 2019). They found a global reduction in circRNAs and aberrant PKR activation in peripheral blood mononuclear cells (PBMCs) in patients with SLE. They also showed that many circRNAs in normal PBMCs tend to form 16–26 bp intra-molecularly imperfect RNA duplexes (intra-dsRNA). These unique structures can bind to PKR, a receptor that can sense viral nucleic acid and direct antiviral activity (Schlee and Hartmann, 2016; Liu et al., 2019). In the early cellular innate immune response, these circRNAs are degraded by RNase L. This releases active PKR, which is in turn linked to SLE progression (**Figure 3F**). In this case, the unique intra-dsRNA structure and the degradation of circRNA are two important factors that inhibit and activate PKR activity, respectively.

## Functions of circRNAs

### circRNAs Serve as Sponges for miRNAs

In recent years, the diverse regulatory mechanisms of circRNAs have become clear, as listed in **Table 2**. To regulate mature miRNA activity some circRNAs serve as miRNA sponges. In human and mouse brains, the natural antisense transcript of cerebellar degeneration-related protein 1 (*CDR1as*) is a circRNA found in the cytoplasm of neurons. *CDR1as* contains over 70 conserved seed matches for miR-7 and one binding site for miR-671 (Jens, 2013). According to preliminary studies, *CDR1as* can absorb miR-7 and the binding sites are only partially complementary to miR-7, which ensures that *CDR1as* is not sliced by Ago2. However, *CDR1as* is almost fully complementary to miR-671, thus the miR-7 cargo can be released by miR-671-mediated slicing of *CDR1as* (Kopp and Mendell, 2018). The deregulation of miR-7 results in the upregulation of immediate early genes (IEGs), which are strongly linked to increased neuron activity (Piwecka et al., 2017) (**Figure 3G**). However, *CDR1as* also functions in non-brain tissues, such as in islet and HCC cells, where it targets miR-7 expression (Xu et al., 2015; Yu et al., 2016), and in bladder cancer tissues where it sponges miR-135a (Li P. et al., 2018). With development of the ceRNA hypothesis, additional circRNAs have been discovered to act as miRNA sponges in cells (Zheng et al., 2016; Shan et al., 2019; Wang et al., 2019).

**Table 2:** The identified circRNAs functions.

Name	Origin	Structure	Function	References
*circHIPK3*	Human	Single exon	miRNA sponge	(Zheng et al., 2016)
*circ-Ccnb1*	Human	Multiple exons	Interacts with protein	(Fang et al., 2018)
*circACC1*	Human	Multiple exons	Scaffold	(Li et al., 2019)
*circMbl*	*Drosophila melanogaster*	Single exon	Regulates maternal gene	(Ashwal-Fluss et al., 2014)
*CircSEP3*	*Arabidopsis*	Single exon	Regulates maternal gene	(Conn et al., 2017)
*FECR1*	Human	Multiple exons	Regulates maternal gene	(Chen et al., 2018)
*ci-ankrd52*	Human	Intronic	Regulates maternal gene	(Zhang et al., 2013)
*ElciEIF3J*	Human	Exon-intron	Regulates maternal gene	(Li Z. et al., 2015)
*circRFWD2*	Mouse	Multiple exons	Pseudogene host	(Dong et al., 2016)
*circPan3*	Mouse	Multiple exons	Protects mRNAs	(Zhu et al., 2019)
*circ-SHPRH*	Human	Multiple exons	Produces a peptide	(Zhang et al., 2018)
*Circ-ZNF609*	Human, Mouse	Multiple exons	Produces a peptide	(Legnini et al., 2017)

### circRNAs Interact Directly With Proteins, Serve as Decoys, or Act as Scaffolds

Similar to linear lncRNAs, circRNAs also have the ability to interact with proteins and regulate protein function (**Figure 3H**). The circRNA, *circ-Ccnb1*, can inhibit breast cancer progression and was enhanced by mutant p53 (Yuan et al., 2006; Fang et al., 2018). The tumor suppressor p53 is a transcription factor that contains 393 amino acids but mutant p53 enhances cancer progression and malignancy. The malignancy consequences that result from the many possible p53 mutations are complicated making the study of downstream p53 signaling difficult. Two key proteins, H2AX and Bclaf1, which function in DNA repair and cell mitosis, play roles in cancer development (Sone et al., 2014; Zhou et al., 2014). Mutant p53 does not bind to H2AX enabling a new approach to repress malignant tumor progression caused by mutant p53. A model of *circ-Ccnb1*-protein interactions was proposed, in which *circ-Ccnb1* interacts with H2AX and wild-type p53 to sustain cell proliferation and survival. However, in the mutant p53 cell (in which *circ-Ccnb1* was down-regulated), ectopically delivered *circ-Ccnb1* interacts with H2AX and Bclaf1, which induces the death of the p53 mutant cancer cells (Fang et al., 2018).

A recent study indicated that circRNAs can serve as decoys that compete with mRNAs in binding to proteins (Abdelmohsen et al., 2017). HuR is a RBP that associates with a wide range of RNAs to regulate protein expression patterns (Lebedeva et al., 2011). HuR positively regulates the translation of Poly(A)-binding protein nuclear 1 (*PABPN1*) mRNA. Interestingly, the circRNA, *CircPABPN1*, which is derived from the *PABPN1* pre-mRNAs, can regulate the translation of its linear counterparts. High levels of *CircPABPN1* can suppress HuR binding to *PABPN1* mRNA, leading to decreased translation. *CircPABPN1* also acts as a decoy to competitively bind to HuR and negatively regulate *PABPN1* mRNA translation (Abdelmohsen et al., 2017) (**Figure 3H**). The CCHC-type zinc finger nucleic acid binding protein, CNBP, binds to the *HuR* promoter and promotes its transcription, which plays an important role in GC. However, *circ-HuR* derived from *HuR* interacts with CNBP and inhibits its binding to the *HuR* promoter, resulting in repression of tumor progression. In this case, *circ-HuR* might also be a decoy to regulate the function of CNBP (Yang et al., 2019).

circRNAs have also been observed to act as scaffolds, promoting protein assembly (Du et al., 2016; Li et al., 2019). AMP-activated protein kinase (AMPK) is a tri-complex consisting of an α catalytic subunit and β and γ regulatory subunits that is a critical sensor of cellular energy status (Hardie et al., 2012). During serum deprivation, *circACC1* derived from *ACC1*-pre-mRNA regulates the assembly of AMPK, promoting β-oxidation and glycolysis. Upon metabolic stress, *circACC1* is up-regulated in cells and interacts with the regulatory β and γ subunits to form a ternary complex, which promotes lipid metabolism (Li et al., 2019). In this case, the circRNA may serve as a scaffold to facilitate the assembly of AMPK and increase its enzyme activity as part of an efficient strategy to cope with environmental pressure (**Figure 3H**).

### circRNAs Protect mRNAs From Degradation

In adult mice, the intestinal epithelium is renewed from multipotent intestinal stem cells (ISCs) and is the most rapidly self-renewing tissue with a turnover of 5 days (Zhu et al., 2019). ISCs located at the base of intestinal crypts are capable of giving rise to all epithelial lineages and exhibit long term self-renewal (Toshiro et al., 2009). Innate lymphoid cells (ILCs) located on mucosal surfaces potentiate the immune system, sustain mucosal integrity and tissue homeostasis (David and Hergen, 2015). IL-13 secreted by ILC2s engages with IL-13Rα1, which is an IL-13 receptor subunit on crypt ISCs, and activates Wnt-β-catenin signaling (Zhu et al., 2019). Interestingly, a circRNA named *circPan3* derived from the *Pan3* gene transcript is highly expressed in mouse and human ISCs. *circPan3* protects *Il13ral* mRNA from KSRP (an mRNA decay protein) mediated degradation and promotes the production of IL-13Rα1 in crypt ISCs, resulting in the reception of more IL-13 (Zhu et al., 2019) (**Figure 3I**).

### circRNAs With Protein-Coding Ability

Although circRNAs lack the 5′ end 7-methylguanosine (m^7^G) cap structure and the 3′ poly(A) tail that are necessary for mRNA translation, they have the potential to produce proteins. In 1995, an artificial circRNA was demonstrated to be translatable in eukaryotic cells (Chen and Sarnow, 1995). Recent studies have shown that specific endogenous circRNAs also code for proteins. To yield proteins, circRNAs need IRES elements, which directly bind initiation factors or the ribosome itself to drive translation of the ORF (Tatomer and Wilusz, 2017) (**Figure 3J**).

The human SNF2 histone linker PHD RING helicase (*SHPRH*) gene, an E3 ligase that targets the proliferating cell nuclear antigen (PCNA) for degradation (Akira et al., 2006; Ildiko et al., 2006), is located in the 6q24 chromosomal region. The loss of heterozygosity in this region is associated with a wide variety of cancers. Interestingly, this region also produces a novel circRNA named *circ-SHPRH*, which encodes a 17 kDa protein, SHPRH-146aa. Interestingly, SHPRH-146aa shares the same amino acid sequence as the C-terminal 1520–1651 residues of full-length SHPRH. The common amino acid sequence of SHPRH-146aa reduces the likelihood of SHPRH degradation by another E3 ligase, DTL. Overexpression of SHPRH-146aa reduces malignancy and tumorigenicity both *in vitro* and *in vivo* (Zhang et al., 2018). Thus, SHPRH-146aa might protect full-length SHPRH from DTL-induced ubiquitination (Zhang et al., 2018).

*circ-ZNF609* is another example of a protein-coding circRNA identified in murine and human myoblasts that specifically controls myoblast proliferation. *circ-ZNF609* originates from the second exon of its host gene and contains a 753-nt ORF from the start codon to an in-frame STOP codon. Sucrose gradient fractionation experiments proved that *circ-ZNF609* binds to polysomes. Using an expression vector and the CRISPR/Cas9 system, a 3xFLAG-coding sequence was inserted upstream of the STOP codon *in vitro* and *in vivo*. Western blot experiments then showed that *circ-ZNF609* has the ability to produce a protein (Legnini et al., 2017).

## Conclusions and Perspectives

RNA-seq has revealed thousands of functional lncRNA molecules in diverse species. The mechanisms of lncRNAs regulation are much more diverse than previously thought. The discovery of functional lncRNAs might shed new light on embryonic development, psychological disorders, and physical diseases. In the past few decades, novel end structures have been shown to play important roles in the functions of linear lncRNAs. With the development of integrated approaches, there is great potential to discover new types of linear lncRNA. lncRNAs seem to perform their functions based on their unique nucleotide sequence, which enables specific binding to DNA, RNA, and RBPs or absorption of miRNAs. Nevertheless, we should not ignore the transcriptional activity and DNA elements of some linear lncRNA loci to influence neighborhood gene expression or the potential of linear lncRNAs to encode peptides.

Nowadays, circRNAs are considered to be effective at regulating cell progression via multiple mechanisms. The study of *CDR1as* revealed that circRNAs regulate mammalian brain function by absorbing miRNAs. This mode of action is also found in other cells, especially cancer cells. Otherwise, the regulatory activities of circRNAs seem to be related to functional RBPs. The interactions between circRNAs and RBPs play critical roles in gene regulation and signal transduction. Remarkably, given the vast number of circRNAs, we still need to learn more about the roles of circRNAs in cells, in healthy and diseased tissues. However, it is difficult to determine circRNA structures because of the large sequence overlap between circRNAs and their linear cognate RNAs (Liu et al., 2019). The development of new methodologies will facilitate progress in this field (Li X. et al., 2018).

Further painstaking work is needed to reveal the detailed molecular mechanisms by which linear lncRNAs and circRNAs regulate biological process. However, the biggest challenge is the transfer of research findings to clinical application and trials. To date, clinical trials of miRNA therapeutics have been conducted based on an extensive body of literature and a simple regulatory model (Janssen et al., 2013), but this is difficult for linear lncRNAs and circRNAs because of the complexity of their structures and regulatory mechanisms. Nevertheless, linear lncRNAs and circRNAs are expected to be subjected to clinical trials in the near future (Chen et al., 2016; Brandenburger et al., 2018; Vo et al., 2019).

## Author Contributions

TQ, JL, and K-QZ conceived this manuscript. TQ wrote the draft manuscript. All authors read and approved the manuscript.

## Funding

This work is jointly funded by the National Natural Science Foundation of China (approved nos. 31970073, 31760538, and 31560025), and the Department of Science and Technology of Yunnan Province (2017HB006).

## Conflict of Interest

The authors declare that the research was conducted in the absence of any commercial or financial relationships that could be construed as a potential conflict of interest.
